# Immune priming and pathogen resistance in ant queens

**DOI:** 10.1002/ece3.1070

**Published:** 2014-04-11

**Authors:** Dumas Gálvez, Michel Chapuisat

**Affiliations:** Department of Ecology and Evolution, Biophore, UNIL-Sorge, University of LausanneLausanne, Switzerland

**Keywords:** formicine ants, immune priming, immunity, life-history, life span, mating

## Abstract

Growing empirical evidence indicates that invertebrates become more resistant to a pathogen following initial exposure to a nonlethal dose; yet the generality, mechanisms, and adaptive value of such immune priming are still under debate. Because life-history theory predicts that immune priming and large investment in immunity should be more frequent in long-lived species, we here tested for immune priming and pathogen resistance in ant queens, which have extraordinarily long life span. We exposed virgin and mated queens of *Lasius niger* and *Formica selysi* to a low dose of the entomopathogenic fungus *Beauveria bassiana,* before challenging them with a high dose of the same pathogen. We found evidence for immune priming in naturally mated queens of *L. niger*. In contrast, we found no sign of priming in virgin queens of *L. niger*, nor in virgin or experimentally mated queens of *F. selysi*, which indicates that immune priming in ant queens varies according to mating status and mating conditions or species. In both ant species, mated queens showed higher pathogen resistance than virgin queens, which suggests that mating triggers an up-regulation of the immune system. Overall, mated ant queens combine high reproductive output, very long life span, and elevated investment in immune defense. Hence, ant queens are able to invest heavily in both reproduction and maintenance, which can be explained by the fact that mature queens will be protected and nourished by their worker offspring.

## Introduction

Researchers have long believed that the immune system of invertebrates lacked memory and specificity (Rowley and Powell 2007). However, recent studies have documented that invertebrates exposed to a low dose of a pathogen became more resistant when later exposed to a high dose of the same pathogen, a phenomenon called “immune priming” (Rolff and Reynolds 2009). Immune priming has been found in insect species exposed to fungi (Rosengaus et al. 1999), bacteria (Sadd and Schmid-Hempel 2006; Pham et al. 2007; Rosengaus et al. 2013), protozoa (Rodrigues et al. 2010) and viruses (Tidbury et al. 2011). However, the generality, adaptive significance, and mechanistic basis of invertebrate immune priming remain unclear. For example, immune priming has recently been documented in ant larvae exposed to bacteria (Rosengaus et al. 2013), but no priming was detected in adult workers of another ant species exposed to fungi (Reber and Chapuisat 2012a), in damselflies exposed to bacteria (González-Tokman et al. 2010), or in pea aphids exposed to bacterial immune elicitors (ter Braak et al. 2013). Moreover, in some cases, immune priming depended on the pathogen tested (Pham et al. 2007).

The persistence and specificity of the immune response may depend on the longevity of the host and the probability of being exposed twice to the same pathogen (Rolff and Reynolds 2009; Best et al. 2013). Overall, life-history theory predicts that long-lived species should invest more resources in maintenance and long-term immunity than short-lived species (Garnier et al. 2013). Along the same lines, long-lived invertebrates are more likely to show immune priming than short-lived ones (Little and Kraaijeveld 2004).

Ant queens are good candidates to show immune priming, because they are extremely long-lived (Keller and Genoud 1997; Jemielity et al. 2005). Queens of *Formica selysi* have been estimated to live for 10–18 years on average (Rosset and Chapuisat 2007; Purcell and Chapuisat 2013) and queens of *Lasius niger* for 20–30 years (Keller 1998; Jemielity et al. 2005). Due to their extraordinarily long life span and sessile life in fixed nests, ant queens are likely to be repeatedly exposed to the same pathogens. Persistent immune protection should thus be beneficial, particularly when considering that queens are essential for colony survival and reproduction.

The investment in immune defense by ant queens may depend on multiple factors and is likely to vary with age and mating status. Young virgin queens arise in the protected confines of their natal colony, where they benefit from group sanitation. Later on, the young queens that found new colonies independently will have to cope with multiple challenges. On the one hand, these queens will be under energetic stress. Typically, they fly away from the natal colony, mate, excavate a new nest, and raise their first offspring in isolation. During this critical period, they rely exclusively on their energy reserves (Hölldobler and Wilson 1990; Cronin et al. 2013). Hence, a trade-off between reproduction and immunity may constrain the investment in immune defenses of founding ant queens. Such a trade-off is common in solitary insects (Zuk and Stoehr 2002; Stahlschmidt et al. 2013). On the other hand, young ant queens may up-regulate their immune response in order to face the challenges of mating, wing shedding, and colony founding in pathogen-rich soil (Baer et al. 2006; Castella et al. 2009; Reber and Chapuisat 2012b). For example, wood ant queens had lower phenoloxidase but higher antibacterial activities after mating (Castella et al. 2009), and leaf-cutting ant queens had higher encapsulation response after digging their burrow, possibly as an adaptive response against pathogens (Baer et al. 2006). In other insects, mating had variable effects on immune investment and pathogen resistance (e.g., Rolff and Siva-Jothy 2002; Lawniczak et al. 2006; Valtonen et al. 2010). It is thus of interest to further investigate the impact of mating on immune resistance of ant queens.

Here, we performed the first tests of immune priming in ant queens and further examined whether immune priming and pathogen resistance depended on the mating status of queens. We exposed virgin or mated queens of two ant species, *Lasius niger* and *Formica selysi,* to low doses of the generalist entomopathogenic fungus *Beauveria bassiana,* and subsequently challenged them with high doses of the same pathogen. We predict that, due to their extraordinary long life span and a key role in the social group, ant queens should exhibit immune priming and high pathogen resistance.

## Materials and Methods

### Collection of queens

**Figure 1:**
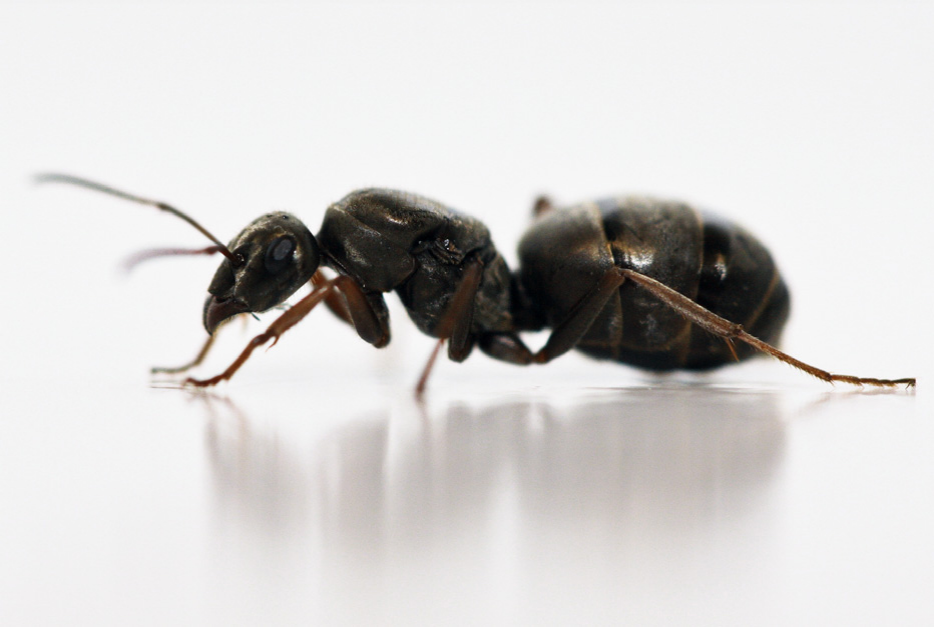
A queen of *Formica selysi*. Photo: Joël Meunier.

We tested immune priming and pathogen resistance in both virgin and mated queens, using naturally mated queens for *L. niger* and experimentally mated queens for *F. selysi* (Fig. 1). We collected mated queens of *L. niger* during two nuptial flights (27 June and 10 July 2012) in the vicinity of the Biophore building on the campus of the University of Lausanne. During this 2-week period, we sampled virgin queens from within nests, either as adult winged queens or as pupae that we kept in the lab until emergence. We performed the experiments with queens that were at least a week old after emergence.

We collected sexual pupae of *F. selysi* between 13 June and 28 September 2012, from a population located between Sierre and Susten along the River Rhône in central Valais, Switzerland (Chapuisat et al. 2004). The males and females emerged in the laboratory, in separate boxes. We controlled mating experimentally. Specifically, we randomly allocated half of the queens to become mated and placed these queens with males in mating boxes, as described in Reber et al. (2010). Only two of 82 queens did not mate in these conditions. The other half of the queens remained virgin. We handled these virgin queens in the same way as the queens that mated, except that no male was placed in the mating box.

### Immune priming and pathogen resistance of ant queens

We used a strain of *Beauveria bassiana* that we had isolated from soil of the *F. selysi* population (Reber and Chapuisat 2012b). This generalist fungal entomopathogen is relatively common in field colonies of *F. selysi* (Reber and Chapuisat 2012b) and is pathogenic to many insects, including *F. selysi* and other ant species (Kafle et al. 2011; Reber and Chapuisat 2012b). *B. bassiana* produces asexual spores (=conidia) that attach to the cuticle and germinate. The hyphae penetrates through the cuticle within 3 days, develops in the hemocoel, kills the insect within 8 days, and produces large numbers of external conidiophores (Reber and Chapuisat 2012a). We cultured the fungus on a malt extract agar medium (MEA) for 5 days at 25°C. We harvested the conidia into water solvent (ddH_2_O containing 0.05% Tween-20).

In order to elicit priming, we exposed virgin or mated queens of both *L. niger* and *F. selysi* to a low dose of live conidia. We adjusted the number of spores to correspond to a LD_1.5_ (the dose causing a 1.5% mortality), which differed between the two ant species. Specifically, we deposited 1 *μ*L of *B. bassiana* solution at a concentration of 8 × 10^4^ conidia/mL on the thorax of each *L. niger* queen and 2 *μ*L of *B. bassiana* solution at a concentration of 2 × 10^5^ conidia/mL on the thorax of each *F. selysi* queen. The control groups consisted of queens treated with a corresponding volume of conidia-free water solvent. Seven days after this initial exposure, we challenged two-thirds of the pathogen exposed and naïve queens with a high dose of the same pathogen, corresponding to a LD_50_. Specifically, the challenge consisted in depositing 1 *μ*L of *B. bassiana* solution at a concentration of 2.7 × 10^6^ on the thorax of each *L. niger* queen and 2 *μ*L of *B. bassiana* solution at a concentration of 2.4 × 10^7^ conidia/mL on the thorax of each *F. selysi* queen. The other third of the queens served as controls and were treated with water solvent. The queens were kept individually in glass tubes, with *ad libitum* water and food. We monitored the survival of queens daily for 15 days.

### Pathogen resistance of naïve *L.* niger queens

Because we could sample many *L. niger* queens, we performed an additional experiment to assess the pathogen resistance of virgin or mated *L. niger* queens that we did not previously expose to low dose of the pathogen. We challenged virgin or mated queens of *L. niger* by depositing 1 *μ*L of *B. bassiana* solution (2.7 × 10^6^ conidia/mL) on their thorax. The control queens were treated with 1 *μ*L of water solvent. We monitored the survival of queens as described above.

### Statistical analysis

We used Cox proportional hazard models to test for differences in survival rates between treatments. To test for immune priming, we analyzed the impact of the initial exposure of queens to a low dose of fungus on their survival when later challenged with a high dose of the same pathogen. We performed separate analyses for each mating status (mated or virgin) and species. When analyzing virgin queens of *L. niger*, as well as virgin or mated queens of *F. selysi*, the date of collection and nest of origin of queens were included in the model as random factors. For mated queens of *L. niger*, the date of nuptial flight and the box in which the queens were kept before the experiments were included as random factors.

To test for the impact of mating status on disease resistance, we compared the survival of mated and virgin queens within control or pathogen treatments. We also analyzed the impact of mating status on pathogen resistance in the additional sample of *L. niger* queens that we did not expose to initial priming. All the analyses were carried out with R 3.0.0, using Cox mixed-effect models as implemented in the package ‘coxme’ (R Development Core Team 2013). We systematically performed Bonferroni–Holm corrections for multiple comparisons, and we report adjusted *p*-values (Holm 1979).

## Results

**Figure 2:**
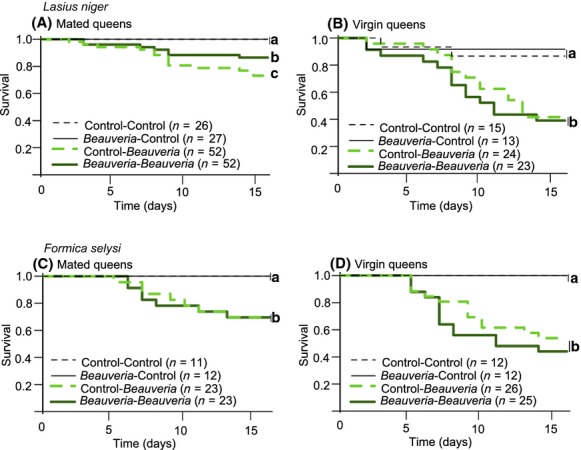
Test of immune priming in queens of *Lasius niger* (upper panels, A and B) and *Formica selysi* (lower panels, C and D). The figure shows Kaplan-Meier survival curves for mated queens (left panels, A and C) and virgin queens (right panels, B and D), respectively. In controls, the queens were exposed to control solvent and challenged with control solvent (Control–Control, black thin dashed line) or exposed to a low dose of the entomopathogenic fungus *B. bassiana* and challenged with control solvent (*Beauveria*–Control, black thin continuous line). In the test of priming, queens were exposed to control solvent and challenged with a high dose of *B. bassiana* (Control–*Beauveria*, light green bold dashed line) or exposed to a low dose of *B. bassiana* and challenged with a high dose of *B. bassiana* (*Beauveria*–*Beauveria,* dark green bold continuous line). Sample sizes (number of queens) for each treatment are indicated in brackets. Different lower case letters indicate treatments that differed significantly from one another.

In all experiments, the challenge with a high dose of the entomopathogenic fungus *B. bassiana* had a negative impact on the survival of ant queens (Fig. 2). Indeed, the fungal pathogen significantly reduced the survival of virgin and mated queens of *L. niger* and *F. selysi*, both for naïve queens (Fig. 2; Control–*Beauveria* vs. Control–Control: mated queens of *L. niger*, *χ*^2^ *=* 32.6, *d.f*. = 1, *P* < 0.001; virgin queens of *L. niger*, *χ*^2^ = 7.4, *d.f*. = 1, *P* = 0.02; mated queens of *F. selysi*, *χ*^2^ = 6.2, *d.f*. = 1, *P* = 0.02*;* virgin queens of *F. selysi*, *χ*^2^ = 12.2, *d.f*. = 1, *P* = 0.002) and for queens that had been previously exposed to the pathogen (Fig. 2; *Beauveria*–*Beauveria* vs. *Beauveria*–Control: mated queens of *L. niger*, *χ*^2^ = 6.4, *d.f*. = 1, *P* = 0.02; virgin queens of *L. niger*, *χ*^2^ = 6.2, *d.f*. = 1, *P* = 0.02; mated queens of *F. selysi*, *χ*^2^ = 6.6, *d.f*. = 1, *P* = 0.02; virgin queens of *F. selysi*, *χ*^2^ = 14.4, *d.f*. = 1, *P* < 0.001). Moreover, virgin queens of *L. niger* that were collected as adults or pupae did not differ in survival when challenged with the pathogen (*χ*^2^ = 0.03, *d.f*. = 1, *P* = 0.85), so we pooled the two types of virgin queens in the analysis.

We found evidence for immune priming in mated queens of *L. niger* (Fig. 2A). Indeed, when challenged with a high dose of *B. bassiana*, mated queens that had been previously exposed to a low dose of the same pathogen showed a significantly higher resistance than naïve mated queens (Fig. 1A; *Beauveria*–*Beauveria* vs. Control–*Beauveria*, *χ*^2^ = 26.2, *d.f*. = 1, *P* < 0.001). In contrast, we did not find evidence of immune priming in virgin queens of *L. niger*, as the exposure to a low dose of the fungus did not increase their resistance to the fungal challenge (Fig. 2B; *Beauveria*–*Beauveria* vs. Control–*Beauveria*, *χ*^2^ = 0.38, *d.f*. = 1, *P* = 0.76).

In *F. selysi*, we found no evidence for immune priming in mated or virgin queens. Naïve and previously exposed queens did not differ significantly in their resistance to the fungal challenge, both for mated queens (Fig. 2C; *Beauveria*–*Beauveria* vs. Control–*Beauveria*, *χ*^2^ = 0.003, *d.f*. = 1, *P* = 0.96) and for virgin queens (Fig. 2D; *Beauveria*–*Beauveria* vs. Control–*Beauveria*, *χ*^2^ = 2.17, *d.f*. = 1, *P* = 0.14).

**Figure 3:**
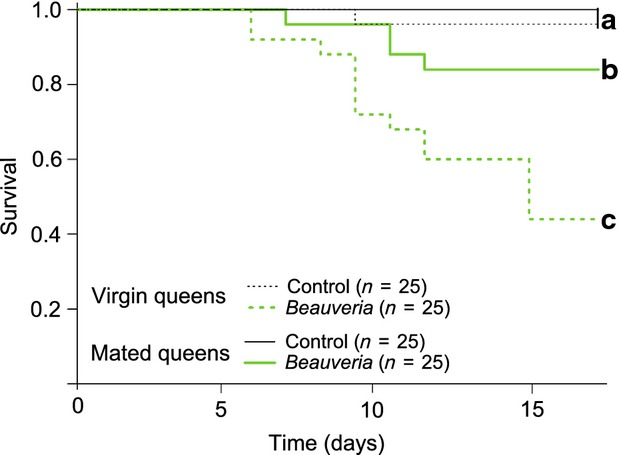
Test of the effect of mating on immune resistance in *L. niger* queens. The figure shows Kaplan–Meier survival curves for virgin queens (dashed lines) and mated queens (continuous lines) that were either exposed to control solvent (Control, black thin lines) or to a high dose of the entomopathogenic fungus *B. bassiana* (*Beauveria*, light green bold lines). No prior exposure (priming) was performed in this experiment. Sample sizes (number of queens) for each treatment are indicated in brackets. Different lower case letters indicate treatments that differed significantly from one another.

In both species, mated queens were more resistant to the fungal pathogen than virgin queens (Fig. 2 and 3). In *L. niger*, this positive effect of mating was observed among previously exposed queens (Fig. 2A and 2B; *Beauveria*–*Beauveria* treatment, mated vs. virgin queens, *χ*^2^ = 13.8, *d.f*. = 1, *P* < 0.001), as well as among naïve queens (Fig. 2A and 2B; Control–*Beauveria* treatment, mated versus virgin queens, *χ*^2^ = 14.0, *d.f*. = 1, *P* < 0.001). In *F. selysi*, because there was no evidence of immune priming, we pooled the data of naïve and previously exposed queens, before comparing the fungal resistance of mated and virgin queens. Overall, mated *F. selysi* queens were more resistant to the fungal pathogen than virgin queens (Fig. 2C and 2D; *Beauveria* challenge, mated vs. virgin *χ*^2^ = 7.1, *d.f*. = 1, *P* = 0.007).

In the additional experiment involving naturally mated and virgin *L. niger* queens that we did not prime, mated queens also showed a significantly higher pathogen resistance than virgin queens (Fig. 3). Again, the fungal challenge with a high dose of *B. bassiana* had a significant negative impact on the survival of both virgin (Fig. 3; *Beauveria* vs. Control, *χ*^2^ = 17.7, *d.f*. = 1, *P* < 0.001) and mated queens (Fig. 3; *Beauveria* vs. Control, *χ*^2^ = 5.8, *d.f*. = 1, *P* = 0.03), and the negative impact of the pathogen was significantly higher for virgin than mated queens (Fig. 3; *Beauveria* challenge, mated vs. virgin queens, *χ*^2^ = 8.53, *d.f*. = 1, *P* = 0.01).

## Discussion

We found evidence for immune priming in naturally mated queens of *L. niger* exposed to *B. bassiana*. However, we detected no sign of immune priming in virgin queens of *L. niger*, nor in virgin or experimentally mated queens of *F. selysi,* when exposing the queens in the same way to the same pathogen. Hence, the occurrence of immune priming in ant queens depends on their mating status and appears to vary across mating conditions or species.

Immune priming should be particularly advantageous for ant queens that are very long-lived, inhabit fixed nests, and found colonies independently in pathogen-rich ground (Baer et al. 2006; Reber and Chapuisat 2012a). Moreover, newborn workers might acquire some pathogen resistance from the queen (transgenerational priming, *e*.*g*., Sadd et al. 2005; Roth et al. 2010; Zanchi et al. 2012) and transmit it to other nestmates (Traniello et al. 2002; Ugelvig and Cremer 2007; Hamilton et al. 2011; Konrad et al. 2012). Individual and transgenerational immune priming have been found in queens of another ground-nesting social insect, the bumblebee *Bombus terrestris* (Sadd et al. 2005; Sadd and Schmid-Hempel 2006). The occurrence of immune priming in mated queens of *L. niger* is in line with this result and suggests that priming may confer long-term protection against fungal pathogens in ant queens. Interestingly, we detected no priming in virgin queens of *L. niger*, which suggests that mating or other changes in queen condition affect the immune system and allow for subsequent priming in this species.

We found no evidence for immune priming in both virgin and experimentally mated queens of *F. selysi*, a species belonging to the same subfamily as *L. niger*, the formicine. Hence, priming varies according to the species, mating circumstances, or queen condition. Naturally mated queens of *L. niger* have participated to a nuptial flight and might be older than *F. selysi* queens that mated in boxes, which might affect their priming ability. However, the absence of immune priming in *F. selysi* queens is in line with our previous results in workers of the same species, where we detected no priming toward the same fungal pathogen (Reber and Chapuisat 2012a). As we used a *B. bassiana* strain isolated from our study site of *F. selysi* (Reber and Chapuisat 2012b), it is possible that the pathogen has co-evolved with its host and thus is able to evade the immune response in *F. selysi*, but not in *L. niger* (e.g., Schmid-Hempel 2009). Whatever the reason, immune priming is likely to depend on many factors such as host and pathogen species, host conditions, pathogen dose, and virulence (this study; Pham et al. 2007; González-Tokman et al. 2010; Reber and Chapuisat 2012a). Hence, variation in the occurrence of priming is both expected and observed, and one should be cautious about drawing general conclusions on the widespread occurrence and adaptive significance of immune priming in insects (e.g., Hauton and Smith 2007).

In both ant species, mated queens were more resistant to the fungal pathogen than virgin queens. The impact of mating on immune resistance was large and consistent, being significant for naturally mated queens of *L. niger* in two independent tests, as well as for experimentally mated queens of *F. selysi*. The experimental approach is important here, as it controls for factors that may correlate with mating status and immune resistance in naturally mated queens, such as age or physical conditions. In line with our findings, several studies have documented that mating or reproduction was associated with greater pathogen resistance in both social and solitary insects (Shoemaker et al. 2006; Valtonen et al. 2010; Schneider et al. 2011).

The higher resistance of mated queens suggests that mating triggers an up-regulation of their immune system (Tian et al. 2004; Baer et al. 2006; Castella et al. 2009). At the mechanistic level, hormonal changes associated with the onset of egg laying, mating wounds, seminal fluid, pathogens transmitted during mating, wing shedding, or muscle histolysis may play a role in starting the cascade leading to immune activation in ant queens (Shoemaker et al. 2006; Kamimura 2007; Castella et al. 2009). In *Drosophila*, ecdysteroids trigger oogenesis and regulate innate immunity (Rus et al. 2013), while sperm and accessory gland proteins activate female immunity (Peng et al. 2005). Hemocytes may also be involved in the response, as they are involved in phagocytosis, correlate with disease resistance, and increase in frequency or activity after mating in various insect species (Sanjayan et al. 1996; Adamo 2004; Baer et al. 2006).

At the ultimate level, the increased immune resistance of mated queens in both *L. niger* and *F. selysi* and the occurrence of immune priming in *L. niger* are likely to increase the success of independent colony founding in pathogen-rich soil. Overall, mated ant queens have very long life spans, high reproductive output, and elevated investment in immune defense (Keller and Genoud 1997; Baer et al. 2006; Castella et al. 2009; Heinze et al. 2013; this study). This combination of life-history traits contrasts with the pattern observed in most solitary insects, which are generally short-lived and have to trade reproduction for maintenance, and in particular fecundity for longevity or immunity (De Loof 2011).

Hence, the question arises how can ant queens invest heavily in both maintenance and reproduction? The answer most likely lies in their social organization. Indeed, in contrast to females of solitary insects, founding queens would not remain alone: They will soon be part of a society, in which they will be protected and nourished by their worker offspring. Hence, the evolution of reproductive division of labor between queens and workers has alleviated the trade-off between maintenance and reproduction: The queens, which play a central role for colony reproduction and fitness, became both long-lived and highly fertile. This is reminiscent of the segregation between germ line and soma in animals, pointing at common principles in the major evolutionary transitions of life on earth (Szathmáry and Maynard Smith 1995).
